# Endoscopic third ventriculostomy (ETV) for treatment of adult hydrocephalus: long-term followup with 163 patients

**DOI:** 10.1186/2045-8118-12-S1-O8

**Published:** 2015-09-18

**Authors:** Albert Isaacs, Geberth Urbaneja, Mark Hamilton

**Affiliations:** 1 University of Calgary, Canada

## Introduction

Treatment of specific patterns of symptomatic hydrocephalus in the adult patient may be accomplished with endoscopic third ventriculostomy (ETV) as an alternative to insertion of a ventriculoperitoneal (VP) shunt or when VP shunt failure occurs. Treatment of hydrocephalus with a VP shunt, while effective, is associated with a significant shunt failure rate that results in VP shunt revision surgery. This review examines a single center experience with ETV to treat hydrocephalus in symptomatic adult patients.

## Methods

Adult patients (>/=18 years) with a diagnosis of hydrocephalus who were treated with ETV in Calgary between January 1994 and July 2014 were reviewed using a clinic database and registry. All patients were treated by one neurosurgeon.

## Results

163 adult patients with symptomatic hydrocephalus treated with ETV were identified (male=92; female=71). Mean age at the time of ETV was 46 years (range 18-83 years). 112 underwent ETV as a primary treatment and 51 patients underwent treatment after presenting with VP shunt failure (secondary ETV). 113/163 patients had a diagnosis of aqueductal stenosis, 22/163 had a diagnosis of tumor. Mean followup was 8.2 years (range 0.3-18.4 years). Symptoms in 149/163 (91.4%) of ETV patients were better or unchanged at last followup. 104/118 (88.1%) of primary ETV patients were shunt free at last followup. 39/45 (86.7%) of secondary ETV patients were shunt free at last followup.

## Conclusions

Endoscopic (ETV) treatment of hydrocephalus is an effective longterm treatment in a select population adult patients with hydrocephalus. Outcome/results are similar for patients where ETV is used as either a primary or secondary treatment. 87-88% of patients remain shunt free with a mean 8.2 years of followup.

## Learning objectives

1) To understand the role of ETV for primary treatment of hydrocephalus in the adult patient.

2) To understand the role of ETV for secondary treatment of hydrocephalus in the adult patient.
